# Digital transformation of mental health services

**DOI:** 10.1038/s44184-023-00033-y

**Published:** 2023-08-22

**Authors:** Raymond R. Bond, Maurice D. Mulvenna, Courtney Potts, Siobhan O’Neill, Edel Ennis, John Torous

**Affiliations:** 1 School of Computing, Ulster University, Belfast, UK; 2 School of Psychology, Ulster University, Coleraine, UK; 3 Department of Psychiatry, Beth Israel Deaconess Medical Center, Harvard Medical School, Boston, MA USA

**Keywords:** Health care, Psychology

## Abstract

This paper makes a case for digital mental health and provides insights into how digital technologies can enhance (but not replace) existing mental health services. We describe digital mental health by presenting a suite of digital technologies (from digital interventions to the application of artificial intelligence). We discuss the benefits of digital mental health, for example, a digital intervention can be an accessible stepping-stone to receiving support. The paper does, however, present less-discussed benefits with new concepts such as ‘poly-digital’, where many different apps/features (e.g. a sleep app, mood logging app and a mindfulness app, etc.) can each address different factors of wellbeing, perhaps resulting in an aggregation of marginal gains. Another benefit is that digital mental health offers the ability to collect high-resolution real-world client data and provide client monitoring outside of therapy sessions. These data can be collected using digital phenotyping and ecological momentary assessment techniques (i.e. repeated mood or scale measures via an app). This allows digital mental health tools and real-world data to inform therapists and enrich face-to-face sessions. This can be referred to as blended care/adjunctive therapy where service users can engage in ‘channel switching’ between digital and non-digital (face-to-face) interventions providing a more integrated service. This digital integration can be referred to as a kind of ‘digital glue’ that helps join up the in-person sessions with the real world. The paper presents the challenges, for example, the majority of mental health apps are maybe of inadequate quality and there is a lack of user retention. There are also ethical challenges, for example, with the perceived ‘over-promotion’ of screen-time and the perceived reduction in care when replacing humans with ‘computers’, and the trap of ‘technological solutionism’ whereby technology can be naively presumed to solve all problems. Finally, we argue for the need to take an evidence-based, systems thinking and co-production approach in the form of stakeholder-centred design when developing digital mental health services based on technologies. The main contribution of this paper is the integration of ideas from many different disciplines as well as the framework for blended care using ‘channel switching’ to showcase how digital data and technology can enrich physical services. Another contribution is the emergence of ‘poly-digital’ and a discussion on the challenges of digital mental health, specifically ‘digital ethics’.

## Introduction

Mental ill health is pervading society and there is evidence suggesting that there was an increase in mental distress during the recent pandemic^1^. Around one in five people experience a mental health problem each year^2^ and around 70% of people with mental ill health are not treated by healthcare personnel^3^. Even if a person is referred for treatment, they could further deteriorate whilst being enlisted on a long waiting list^4^. These challenges call for service improvements and new efforts to help improve mental health provision. Digital technology could potentially help in providing novel digital interventions that are available 24/7; however, we should avoid having an attitude of technological solutionism^5^ i.e. assuming that ‘digital’ can fix all problems.

What is digital mental health? It is the application of digital technologies in mental healthcare which can be used for many purposes, including mental health and wellbeing promotion and prevention, wellbeing maintenance/self-care, early intervention, or for treating specific mental illnesses using, for example, online video communication technologies. Digital technologies may help by (1) optimising current services by using technologies to create a better, more fluid, user experience, (2) to generate more useful and actionable data for service providers and therapists which can be used to deliver more data-informed ‘personalised’ services (here we refer to service providers as the organisations that deliver a service and the therapist as the person delivering psychotherapy), and (3) to provide new digital interventions for prevention or to deliver or support treatment. It is, however, very important for these digital technologies to be co-developed with stakeholders and with the user’s requirements at the centre of the design process, as opposed to the design being disproportionately driven by the developers, i.e. computer scientists. There is a wonderful tongue-in-cheek quote from Bigham (www.cs.cmu.edu/~jbigham/), stating that, “*The two hardest problems in computer science are: (i) people, (ii), convincing computer scientists that the hardest problem in computer science is people, and, (iii) off by one errors*”. This quote points to the fact that it is not always the technical aspects of a digital system that is the main challenge, but rather designing the user interface to meet the needs of people as well as optimising the user interface for user retention and engagement. Hence, we need to design digital systems with an in-depth understanding of the end users. This revelation is most evident when observing human behaviour whilst people use any new technology. It can also be well argued that digital mental health requires many interdisciplinary researchers to provide solutions that are comprehensive, usable, and responsible.

## Paper overview

This paper starts by defining digital mental health and presents a suite of technologies to help illustrate the wide range of digital tools that can be used to enhance mental healthcare. This is followed by a discussion about the benefits of digital interventions. In particular, we emphasise the benefit of digital interventions being more anonymous and the fact that these interventions can act as a ‘destigmatising’ and anonymous stepping stone to traditional face-to-face services (in addition to augmenting face-to-face services). We also discuss the benefit of using digital data to provide a more data informed service that can be tailored based on deeper real-world insights into the client’s mental wellbeing. The paper then presents the challenges of digital mental health with a particular focus on digital ethics. For example, digital technology could be perceived as replacing traditional services whilst promoting screen-time. The paper then concludes with an emphasis on stakeholder-centred design as one method to taking a systems thinking approach to addressing many of the challenges in digital mental health transformation.

## A suite of digital mental health technologies

**Fig. 1:**
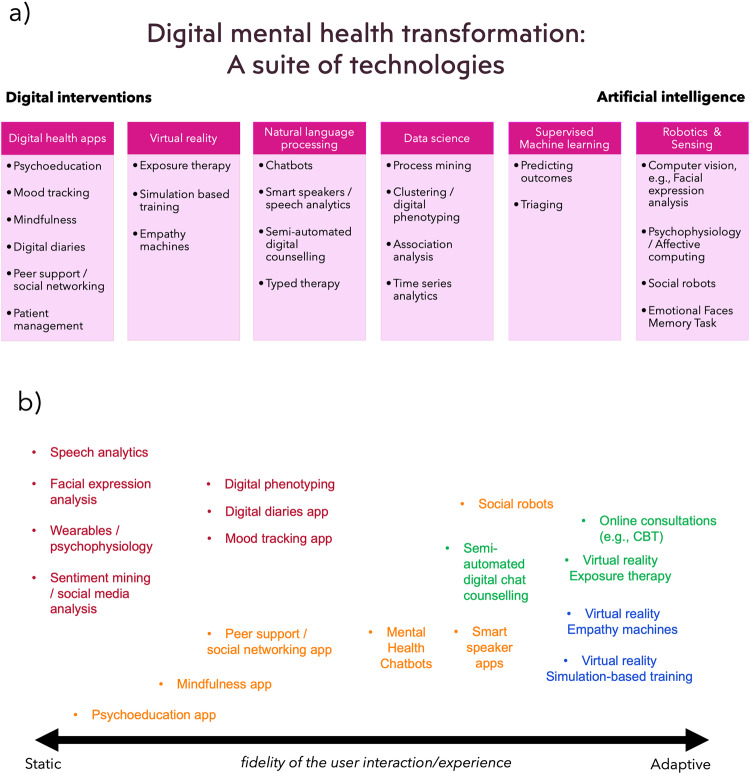
An illustration of the range of digital technologies that can be used in mental health services. **a** shows a suite of digital mental health technologies with example applications, and **b** a notional continuum of digital mental health tools/applications that are relatively static (e.g. a psychoeducational app) as well as those tools that are arguably more interactive and dynamic (e.g. chatbots and virtual reality). In (**b**), the orange labels represent ‘wellbeing support’, the red labels represent ‘monitoring’, the green labels represent ‘treatment’ and the blue labels represent ‘training’.

Different stakeholders might understand the concept of digital mental health from different perspectives. For example, one might think of it as the design of digital interventions (or digital preventions/postventions [preventions can be interventions that prevent mental ill health and postventions can perhaps be interventions that help manage the wellbeing of people after/post a suicide event to provide support during bereavement]) such as smartphone apps or virtual reality (VR), whilst others might be more focussed on the application of artificial intelligence (AI) or digital phenotyping^6^. Given that there may be many perspectives, Fig. 1a presents a suite of digital mental health technologies ranging from digital interventions to the use of AI—although AI can certainly be a part of a digital intervention too. One end of the diagram presents digital health apps that can be used for psychoeducation, mood logging and to aid positive mental health activities such as breathing exercises, mindfulness and gratitude diaries. Apps are the best known format for a digital intervention; however, virtual reality (VR) and augmented reality (AR) can also be used to alleviate phobias using exposure therapy^7^. For example, a client with claustrophobia can be gradually exposed to higher fidelity scenarios, such as initially being exposed to a large elevator and gradually providing exposure towards a smaller elevator and then perhaps to an elevator that includes other people). The benefit of VR is that the environment and the variables are fully controlled. VR can also be used to engender empathy by simulating what it is like to be in someone else’s shoes so to speak (referred to as VR empathy machines^8^). Of course, VR can also be used for simulation-based training to train healthcare professionals in treating clients under different controlled scenarios^9^.

Nevertheless, moving towards AI in the diagram includes natural language processing (NLP). This can include the use of chatbots for typed therapy to allow users to converse about mental health^10^ or using speech analysis to ‘implicitly’ track mood or indeed the use of algorithms that recommend high quality responses for psychotherapists to select and use when delivering online chat-based cognitive behaviour therapy (CBT)^11^. Moreover, a current EU project (called MENHIR—Mental health monitoring through interactive conversations) looks to develop conversational systems to support people living with mental ill health and have undertaken user requirements research to inform the design of NLP applications^12^.

Moving further along this diagram presents the use of data science and machine learning (ML). These technologies allow researchers and service providers to understand their clients and elicit new insights into mental health problems which can lead to service improvement. For example, clustering has been used to discover the types of callers in a crisis helpline^13^, and digital phenotyping^14^ has been used to analyse smartphone usage and activity data to infer mental health states. Supervised ML can also be used with mental health data to automatically predict client outcomes^15^ or for triaging. For example, our work has included the use of ML to predict the type of caller a person will become, in terms of their call behaviour, based on their activity early on in their use of a crisis helpline^16^. This use of ML with helpline data could help triage calls to those who are likely in a crisis. The diagram ends with other advanced digital technologies such as social robotics and computer vision which includes facial expression analysis and affective computing to infer emotions, which are not discussed in this paper. Figure 1b presents a similar set of digital mental health technologies showing those applications that are relatively static (e.g. a psychoeducational app) as well as those tools that are arguably more interactive and dynamic in nature (e.g. chatbots and virtual reality).

## Benefits of digital interventions

**Fig. 2:**
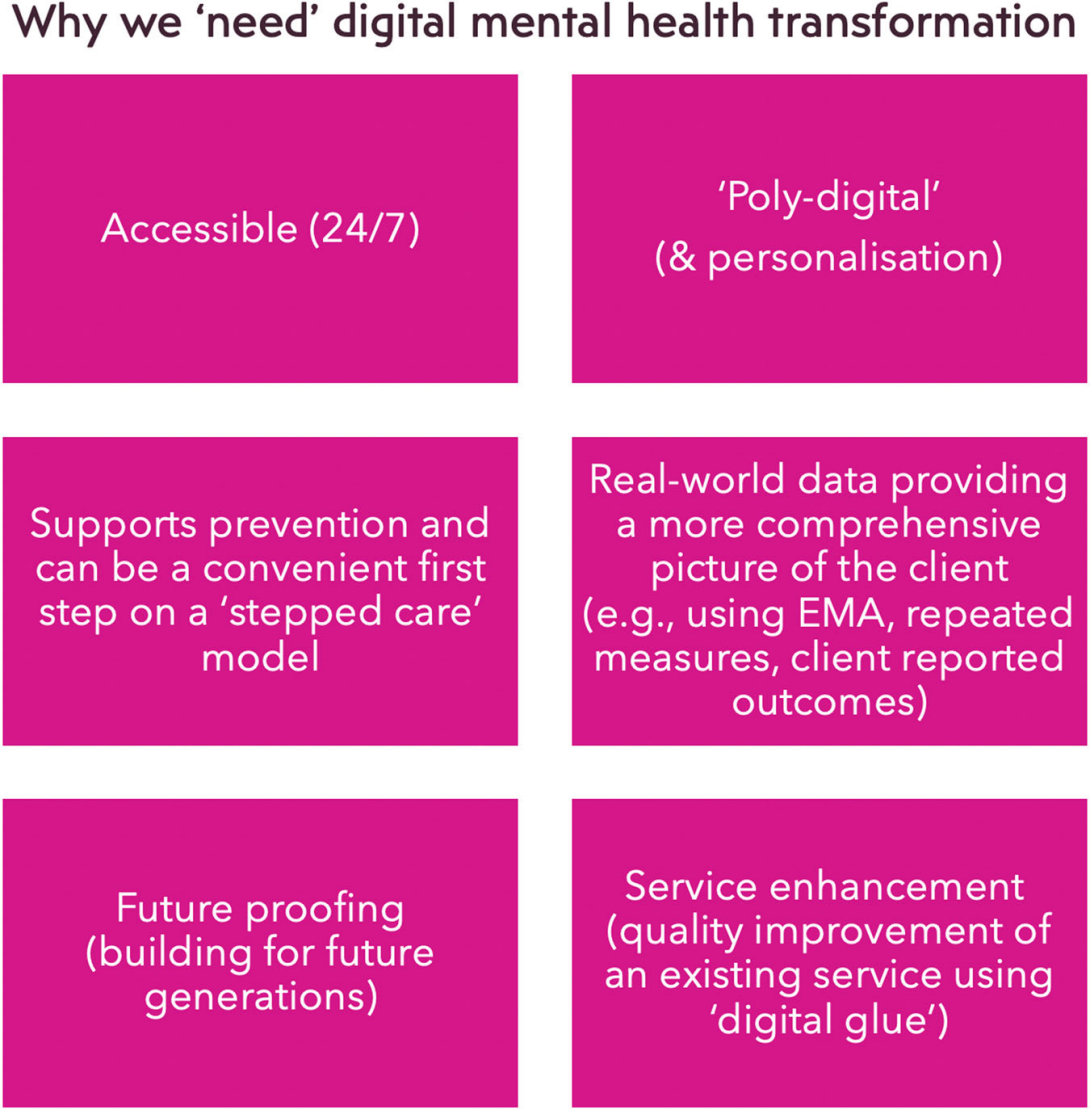
The benefits in digital mental health.

We will discuss those benefits that are presented in Fig. 2. A clear benefit of digital interventions (e.g. apps and chatbots) is that they can have an adjunctive use in therapy provision, and are available 24/7 allowing clients to access support in-between face-to-face therapy sessions and seek support in less sociable hours. However, a less-discussed benefit that is not emphasised enough is that some users may actually prefer to engage with digital support in the first instance and use ‘digital’ as a stepping stone for gaining confidence to step-up onto traditional services (e.g. talking therapies). This preference could be due to the level of anonymity that digital support provides allowing the client to avoid any stigma whilst mitigating the risk of feeling judged in a human facing service^17^, as well as the agency to the individual offered by self-managed care. This idea is revisited later in this paper in the section related to digital ethics.

Another understated benefit is that the digital transformation of mental health provides future proofing for the new generations of citizens who are more acquainted with digitally enhanced services. Mental health problems and disorders often develop during adolescence (1 in 5), and it is important that there are early interventions (perhaps even digital interventions) to help mitigate against further escalation^18^. The younger generation has been coined ‘generation mute’^19^ given that they are more likely to use smartphones to text rather than making phone calls, which could be aligned to the adoption and acceptance of typed therapy using chat-based CBT or chatbots. Younger generations may continue to expect most services to have a digital dimension, and indeed be ‘digital-first'. With this in mind, even the use of digital technologies that are less ‘intervention’ based can be the ‘digital glue’ that can enhance the quality of established services. A good anecdotal example from the fitness industry is the PureGym app (www.puregym.com/app/) which acts as a kind of ‘digital glue’ to enhance the user experience of a gym. The app allows users to book workout classes on the app which can be added to their digital calendar. The app also allows users to exercise from home when needed using ‘digital classes’ aided by high quality video. Moreover, the users can plan their workouts and follow different programmes that can be supported using digital animations with visual instructions and digital timers for each exercise. Finally, the app also uses QR codes to allow users to conveniently enter and exit the gym. This is a good example of how ‘digital glue’ can enhance an established service without replacing it. Moreover, the convenience of using an app to book sessions in advance requires little effort and can perhaps act as a ‘gateway habit’^20^ that leads to greater engagement from the client.

## Poly-digital

**Fig. 3:**
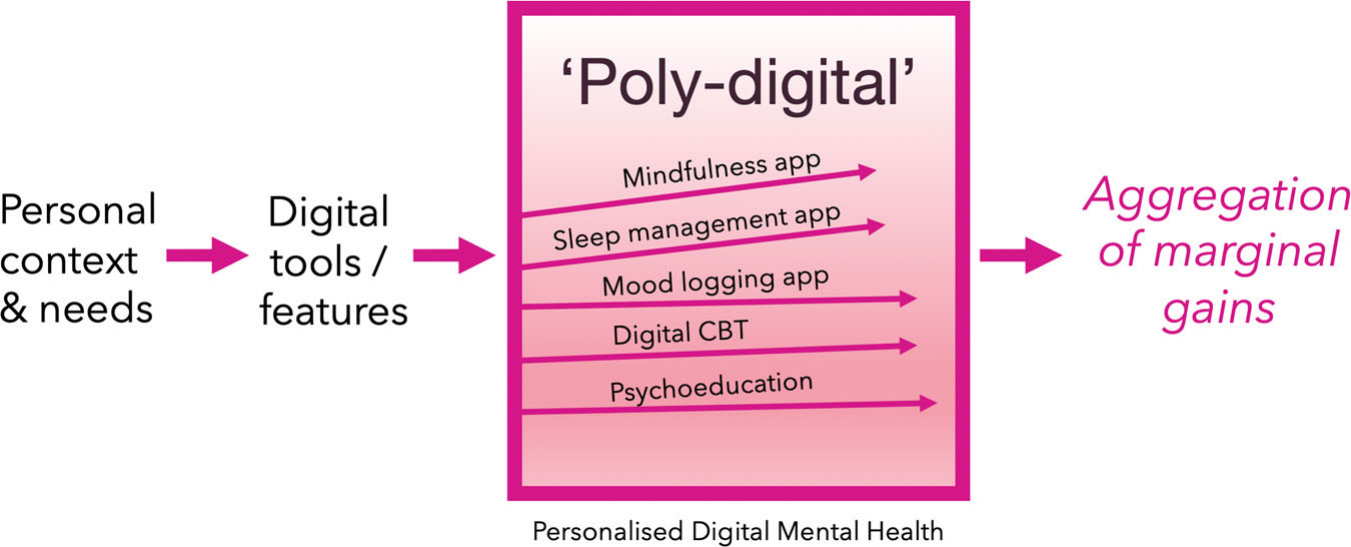
A visual example of ‘poly-digital’. This is where various digital tools are used to manage different aspects of one’s mental wellbeing which can lead to an aggregation of marginal gains. This figure is an example of the myriad of digital tools that a user could be prescribed to use based on their bespoke needs. These digital tools could be different apps or indeed one app that has these different features enabled whilst other features might be disabled by a service provider.

A less-discussed benefit is that a personalised collection of different digital interventions can be used together to help improve various aspects of an individual’s mental wellbeing needs. This is important given that mental wellbeing can be multifactorial and that different symptoms or even the same symptom can be addressed by a collection of diverse digital tools that are all available 24/7. This is what we call, ‘poly-digital’ (Fig. 3), which is the idea that different digital tools can be prescribed based on an individual’s needs (i.e. personalised healthcare). For example, a sleep app can be used to improve sleep hygiene whilst simultaneously using a mindfulness app and a mood logging app to improve a positive mindset. Whilst each app might only have a marginal effect on a person’s mental wellbeing, the aggregation of marginal gains^20^ could perhaps compound to a large effect on one’s overall wellbeing. Indeed, recent data in 2022 suggests that when people use mental health apps, they use several apps and create their own digital toolkits by creating their own poly-digital ecosystem^21^. While it may be challenging to assess the impact of any one app (or part of that app), understanding how people naturalistically engage with apps offers new opportunities to better assess their impact and develop more valid measures to represent their utility. Given the global scope of mental health challenges, even a 1% improvement in say depression symptoms would have a profound impact.

## High resolution client data

**Fig. 4:**
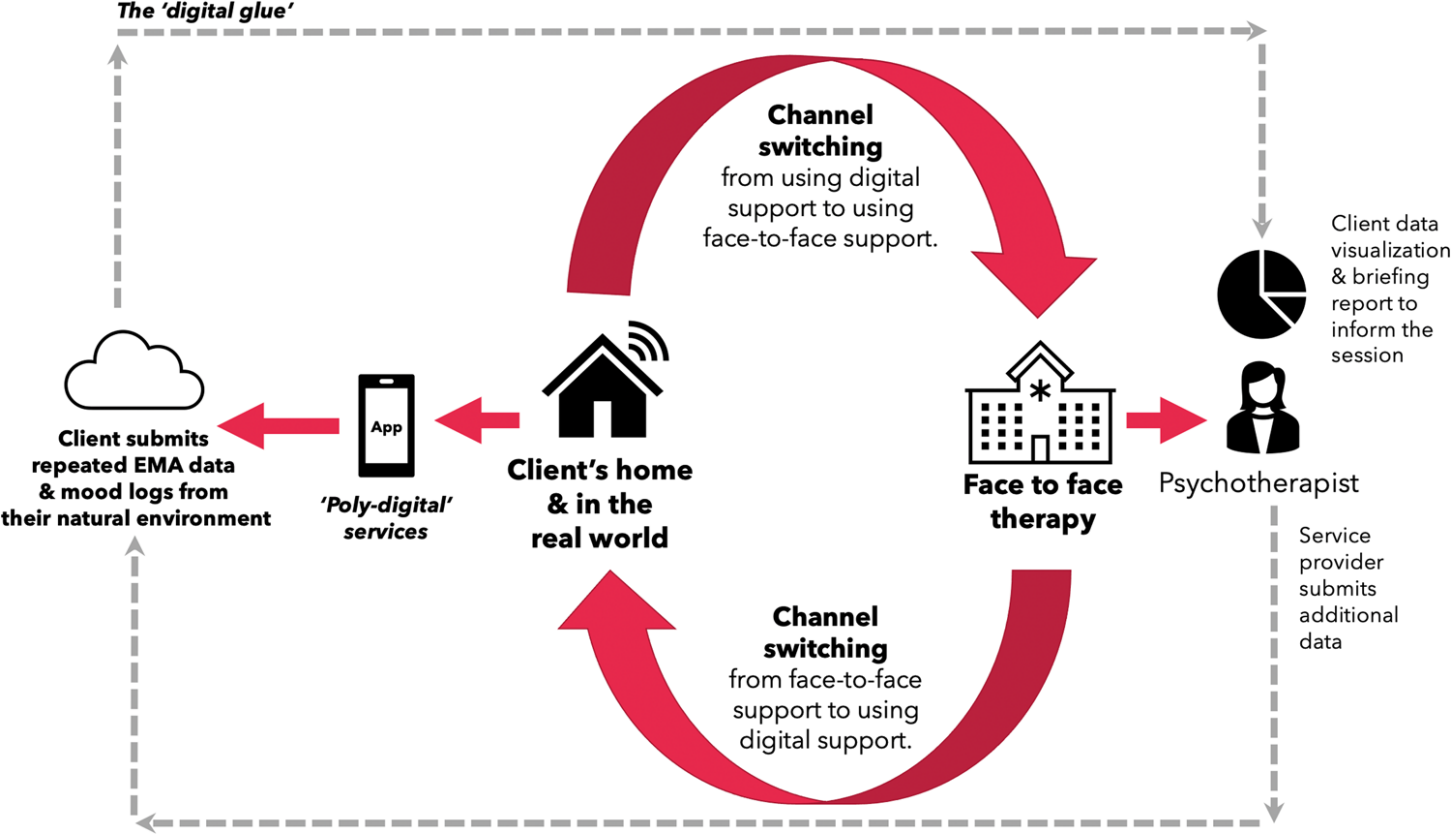
An illustration of blended services/adjunctive therapy. This shows how a service can blend the use of digital support (and digitally curated repeated measures/EMA data) along with traditional physical face-to-face support and provide a higher quality service that is more integrated and ‘data-informed’. This diagram also illustrates the idea of ‘digital glue’ to enhance a current service by cementing the non-digital (physical) and digital support (client self-managed ‘channel switching’ across cyber, human and physical resources).

An under-realised benefit of digital mental health is the amount of ‘useful’ and ‘actionable’ real-world personal health data that can be generated from clients via digital interventions. Today, a number of traditional mental health services may only collect data from clients at a very low sampling rate where mental health scales are, for example, used at monthly talking therapy sessions. Digital apps in particular would allow a user to submit repeated measures at different times of the day and on different days from their own environment. This rich data is also known as ecological momentary assessment (EMA) where single item scales are typically collected in the form of pop-up questions within an app^22^. EMA data provide a high degree of ecological validity as questions are answered ‘in the moment’. This avoids recall bias and may be more useful to healthcare professionals in comparison to mental health scales which may be only ever administered in face-to-face psychotherapy sessions and rely on individuals answering questions retrospectively that are based on their feelings over the previous 2 weeks. Figure 4 illustrates a client ‘channel switching’ between digital and physical support channels, and how EMA data can enhance traditional services. This figure illustrates a framework for blended care in mental health.

Apps can also support the capture of sensor data which can provide real time data on relevant behaviours like sleep duration, exercise levels, tone of voice, etc. This combination of more accessible survey data and real time sensor data opens a new paradigm for digital assessment that is sometimes referred to as digital phenotyping. This type of data allows a service or therapist to gain greater insight into the client’s patterns of mood and condition, which can perhaps better direct a more productive therapy session. A benefit is that the data is collected in the real world within the user’s natural ecology. Having repeated measures of a users’ mental health and mood patterns may enhance the quality of mental health services, but like any data it must be high quality. Just as we would not expect a radiologist to interpret a low-resolution X-ray or an X-ray with lots of missing pixels, today many digital phenotyping signals are still noisy and with missing data and we have much to learn about their full potential.

To revisit the concept of digital glue which could be considered a digital platform that simply connects the client’s use of digital interventions and their real-world data with their therapist and the recommendations from face-to-face services. If such a platform is used, perhaps mental health services could be more efficient. For example, if a digital platform indicates that a client is engaging with digital wellbeing activities and that their data indicates a substantial improvement in their wellbeing, then perhaps this kind of knowledge can enhance the scheduling and frequency of face-to-face sessions. In this sense, a data rich digital platform could inform triaging and optimise operations.

## Challenges in digital mental health

With these benefits, digital mental health is not without profound challenges. Thousands of mental health apps are available and can be readily accessed by the general public, however only a small proportion of these have been assessed and accredited by organisations such as the Organisation for the Review of Care and Health Apps (ORCHA) or the M-Health Index and Navigation Database (MIND). ORCHA is a UK-based organisation that assesses key aspects of health apps, including user experience, data privacy and aspects related to the app’s clinical assurance. A report by ORCHA^23^ detailed that only 32% of available mental health apps would pass a quality assurance benchmark score. A 2022 paper which looked at 578 mental health apps indexed in MIND and rated across 105 dimensions revealed that few apps offered innovative features and many represented privacy risks to users^24^.

This provides a challenge for regulating and quality assuring digital mental health apps. Moreover, there is the known challenge of user retention with digital interventions. For example, the percentage of young people who complete a digital mental health intervention can be as low as 29.4%^25^. The majority of users of mental health apps simply download and delete an app without using the app for any sustained period of time^26^. There are perhaps many speculated reasons for this, e.g. app fatigue, not meeting expectations, the app lacking engaging features or simply the app was not as usable and useful as the user initially thought^27^. Recent and ongoing research underscores that there is no single reason why people cease to engage with apps, and increasingly solutions are focusing beyond simply adding ‘better’ design to considering system wide implementation facilitators and barriers^28^. An overlooked barrier is the lack of clinical training or implementation support to ensure that new digital interventions can seamlessly fit into busy mental health services^29^. Designing the next wave of innovations to serve not only patients, and not only clinicians, but the partnership between both parties may offer a tangible solution to meaningfully improve engagement. Social prescribing may offer some remedy in supporting engagement with digital mental health services, beyond current use in addressing long-term health conditions^30^. Beyond these challenges, there are also many dilemmas related to ‘digital ethics’ in mental health.

## Ethics in digital mental health

**Fig. 5:**
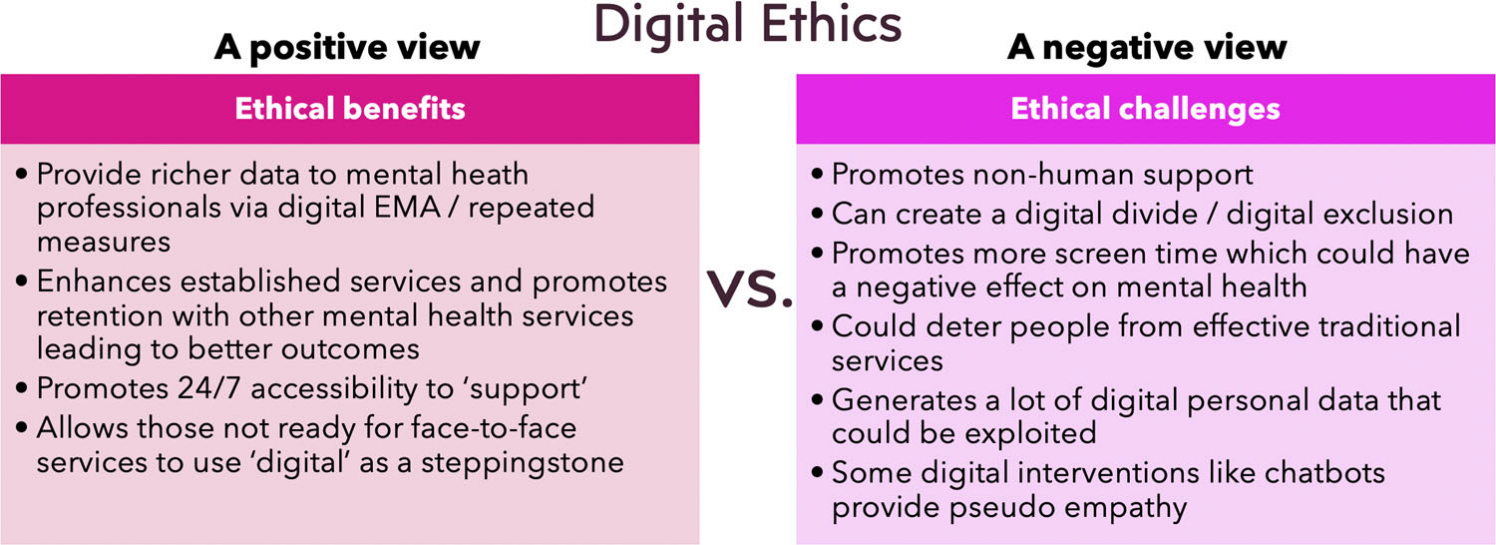
Digital ethics in digital mental health. This figure presents a number of the ethical obligations to develop a digital mental health paradigm and a number of the ethical challenges that need to be addressed.

Figure 5 provides an overview of some of the ethical challenges and ethical benefits. A key ethical challenge is that digital interventions could be perceived as a replacement for human facing services (akin to AI anxiety) and as another digital service that promotes even more screen-time. It is clear from both the patient and clinician perspective that digital interventions should not be used to replace high quality face-to-face services with a computer. While the relationship between screen-time and mental health is complicated^31^, there is stronger agreement that the most effective use of digital interventions is when they are augmented and supported by people^32^.

Whilst there is a role for most forms of technology, each bring their own nuanced ethical challenges. For example, chatbots that allow users to converse with a computer bring their own ethical dilemmas. A chatbot could perhaps, in theory deliver psychotherapy, but a chatbot only provides a kind-of ‘pseudo-empathy’ and does not actually care about the end-user. Hence, should anthropomorphism be avoided or toned down in mental health chatbots? And whilst chatbots can disclose their lack of ability or ‘real’ intelligence (to help calibrate the user’s expectation) and provide a fallback message to signpost users to helplines/human support, this does not guarantee that users won’t even subconsciously anthropomorphise the chatbot and have greater expectations given the fact that it is a humanised natural language interface (refer to the computers as social actors theory^33^). For example, engaging in a ‘conversation’ is an intelligent interaction yet the chatbot discloses that it is not that intelligent (hence there could be a cognitive dissonance between the user’s intuitive expectation of what a conversation is and the lack of technical competence to fulfil this expectation—regardless of the chatbot’s disclosure). Another challenge is that the advice and recommendations that a chatbot provides could also be untested and over-trusted given that the consultation is delivered in the form of a ‘humanised’ dialogue. Bickmore et al.^34^ have presented case studies where smart speakers could potentially provide harmful medical advice. This is, perhaps, in contrast to information seeking when a user uses a web search engine—where users have some autonomy and choice over which website to trust and read. It is also very difficult to quality assure and regulate an AI chatbot given that all possible conversations will not likely be pre-assessed. The challenge of assessing the quality of AI chatbots that generate personalised responses on the fly is due to the fact that the number of dialogue permutations can be very large and it would be almost impossible to assess every possible dialogue. Thankfully, most health related chatbots actually use fixed finite state conversational design (i.e. transparent, pre-defined branching tree logic) and not AI^35^. Moreover, one could argue that the idea of humanising technology in the form of a chatbot is best avoided given that anthropomorphism could induce a level of misunderstanding where users may feel that they are talking to a human even when they are informed that they are conversing with computer (see Computers as Social Actors theory^33^). From this perspective, perhaps we should keep computers as computers and humans as humans, and recognise their complementary strengths (e.g., computers are good with numbers and humans are good with words, and that’s that!, refer to Moravec’s paradox^36^).

Regardless of these ethical challenges, mental health chatbots have become somewhat topical. For example, the Wysa chatbot^37^ is a mental health chatbot that has recently been integrated into a traditional NHS service^38^. Fiske et al.^39^ present studies and instances where clients prefer to ‘chat’ to a digital agent to avoid judgement or embarrassment. Our own recent mental health chatbot called ChatPal focused on some of these ethical issues by (1) avoiding over anthropomorphising the chatbot by not giving it a human name in order to help avoid users from equating the chatbot to human support, (2) disclosing the capability of the chatbot upfront (ensuring users know that the chatbot is not that intelligent), (3) by providing fallback messaging with referrals to human support (crisis helplines), and (4) ensuring that the dialogue scripts were designed by healthcare domain experts and (5) by limiting the AI/NLP capabilities to mitigate the chatbot from generating harmful utterances.

Having discussed the challenges and ethical concerns in digital mental health, many of these challenges should be addressed using methods that involve all stakeholders. By stakeholders, we don’t just mean users. Stakeholders can involve computer-scientists, human-computer interaction (HCI) researchers, policy makers, service designers, service providers, healthcare staff and users.

## Stakeholder-centred design

Researchers such as Thimbleby et al.^40^ have well-articulated that poorly designed digital health products and medical devices can result in medical errors where the user is somewhat misguided by the poor design itself. This can be referred to as a ‘use error’ as opposed to a ‘user error’ since the latter terminology attributes all causation to the user and not to the poor design. This work highlights the need for an effective design process in digital health. A quote often attributed to Henry Ford, *“If I had asked my customers what they wanted they would have said a faster horse”*^41^. This quote illustrates that users are not necessarily inventors or designers. However, user involvement in product design is certainly important. Involving users in the design process is typically referred to as user-centred design (UCD) or human-centred design.

UCD is an approach that positions the user at the centre of the design process^42^. UCD has been successfully used in many product and service designs and is supported by standards^43^. An objective of UCD, is to learn what product or service is best suited to meet the needs and preferences of the user. There are also fresh approaches and arguably the most interesting is the lead user concept^44^. This concept stems from research findings that is perhaps unlike the approach of Henry Ford, given that it is often the user who can realise a commercially successful product or service, rather than the producers, and that a particular type of user, the ‘lead user’, may be responsible for the majority of the innovative thinking^45^.

Another approach that facilitates stakeholder-centred design are living labs. The architect and academic, William J. Mitchell, created the concept of living labs. Mitchell, based at MIT, was interested in how city dwellers could be actively involved in urban planning and city design^46^. Living labs are *“collaborations of public-private-civic partnerships in which stakeholders co-create new products, services, businesses and technologies in real life environments and virtual networks in multi-contextual spheres”*^47^. How living labs actually work centres on methods, processes and services utilised to translate the philosophy into engagement, and these can be summarised as ideation, co-creation, exploration, experimentation and evaluation^48^.

Nielsen^49^ pioneered the concept of usability engineering and describes usability using five concepts, namely, the learnability, efficiency, memorability, errors and satisfaction of a system. Nielsen^50^ also developed ten heuristics or design principles that can be used to guide the design and optimise the usability of a product. The concept of optimising the user experience goes beyond usability and includes concepts such as the look and feel of a product, it’s desirability and perhaps it’s delightfulness. A product that provides an optimal user experience is one that is useful, usable, satisfying to use, perhaps desirable and one that typically matches our mental models allowing the user to interact using ‘system 1 thinking’ (our fast intuitive system of thinking^51^). To optimise the user experience of a product, it is important to involve users in all parts of the design process. Involving users in the requirements elicitation stage can be done via open discussions. Co-designing prototypes and ideating can be referred to as a living labs methodology, participatory design, or co-creation. Of course, these workshops can be extended to hackathons and datathons that involve users. Nevertheless, these approaches should create empathy for the end users (empathy for their needs and their context of use). These workshops can involve many activities, such as anonymous voting using digital polls, co-wireframing different user journeys, design fiction and interactive activities such as card sorting (a useful technique that allow users to create a hierarchy of features in accordance to what is the most and least important to them which can be used to prioritise requirements for a minimum viable product and/or to optimise the visual hierarchy of user interfaces, etc.).

Vial et al.^52^ recently carried out a review on human-centred design methods that have been used to design digital mental health technologies. They found 22 studies that utilised a human-centred design approach, and 27% of the studies that were investigated did not describe any methodology for human-centred design. This 2022 review suggests that there could be a lack of human-centred design in digital mental health and the involvement of users throughout the design process.

Whilst being user centric in terms of focussing on the user’s needs is critical, products do not just live in the user’s hand, but they often live within complex ecosystems, requiring a systems thinking approach which includes involving not just the user but all stakeholders in the design. Martin^53^ describes this in terms of the modern capsule-based coffee machines that provide a great user-centred design but may not consider other stakeholders such as environmentalists. To illustrate the need for stakeholder-centred design in mental health, we could develop a digital technology to address the needs and wants of clients (being only one stakeholder); however, there may be occasions when users want a solution that would not be endorsed by other stakeholders, i.e. mental health experts. For example, one study surveyed mental health experts^54^ regarding their attitudes towards the prescription of mental health chatbots. The study found that mental health experts would support mental health chatbots that help with self-management and psychoeducation, but mental health experts would be less likely to endorse chatbots that deliver ‘treatment’ or provide ‘diagnostics’. Hence, even if users wanted a diagnostic chatbot, a stakeholder-centred perspective would prohibit this from being developed. The authors then used this research to develop ‘ChatPal’ which is described as a chatbot that people needed, but also one that is endorsed by professionals and a chatbot that uses technologies which computer scientists regard as being reliable (so as to avoid harmful digital conversations).

## Discussion and conclusion

This paper is a perspective paper that is mostly based on our own research and reflections. There are many digital technologies that can be used but we need a reliable and universal quality assurance framework to help moderate the delivery of various digital mental health technologies. Organisations such as ORCHA^23^ assess the quality of health apps using a detailed review process resulting in a score out of 100. However, this review process is not specific to mental health and mainly includes the assessment of apps, whereas there is a need to quality assure other mental health technologies such as VR exposure therapies. Mental health applications may require its own set of quality assurance guidelines given the nature of the domain and the risk (e.g., a chatbot giving harmful advice). Moreover, there is a need to quality assure the digital literacy training of therapists as well as the actual integration of a digital platform. For example, we could also quality assure the data pipeline for analysing the real-world data from digital mental health interventions. Furthermore, we could also quality assure, and perhaps even standardise how this real-world data is visualised for therapists to interpret and use to inform their client sessions. This is akin to what we discussed in a previous paper pertaining to the concept of an ‘affectogram’^55^.

A key challenge for the digital transformation of mental health is ‘change management’. It can be a challenge for people to adopt new innovations, even when new innovations will empirically optimise workloads and improve the wellbeing of clients (this is referred to as the ‘baby duck syndrome’ where users can cling onto the first solution that they encounter and struggle to let go—even when their legacy technologies are sub-optimal). For example, one can encounter people queueing at an airport to check-in manually whilst a convenient automated check-in desk is available close by. Given that we are a people of habit, we can sacrifice quality, efficacy and time - just to use the technology that we already know. We arguably spend a disproportionate amount of time on technology development and very little time on digital literacy training and working on adoption challenges with the actual intended end-users. The balance between ‘working with technology’ and ‘working with people’ needs to change. To quote Freeman^56^, *“…projects are about 5 percent technology and 95 percent change management*”.

To summarise all that has been discussed in this paper, the following is a list of recommendations and closing statements:

- Digital technologies can be used to augment existing mental health services without replacing them, and act as a kind of ‘digital glue’ to improve the user experience and future proof services for future generations.
- Digital technologies can be used to facilitate the collection of high quality data and repeated measures (both digital phenotyping and EMA) from clients outside of therapy in order to better inform the service provider and to improve the quality of the time spent with the client face to face.
- Digital mental health interventions can increase the accessibility of support (24/7) and can also be used as a ‘non-stigmatising’ and ‘anonymous’ stepping stone to receiving support.
- A digital mental health platform could provide a personalised set of apps or features (creating a tailored poly-digital ecosystem) to an individual which could collectively result in an aggregation of marginal gains.
- Be sure to consult all stakeholders in the design of digital mental health technologies, for example, we should consider the client needs, the reliability of the technology and the endorsements of the healthcare professionals.
- Be sure to consider all ethical aspects of deploying different kinds of digital technologies in mental health.

### Reporting summary

Further information on research design is available in the Nature Research Reporting Summary linked to this article.

### Supplementary information

Reporting Summary
